# Polyurethane-based foam composites: synthesis, structural characteristics, and radiation shielding properties

**DOI:** 10.1038/s41598-025-95497-7

**Published:** 2025-04-10

**Authors:** Hussein Oraby, Ghada E. Hegazy, Soliman M. ElTalawy, Islam M. Nabil

**Affiliations:** 1 Department of Chemical Engineering, Military Technical College, Cairo, Egypt; 2 National Institute of Oceanography & Fisheries, NIOF, Alexandria, Egypt; 3 Physics Department, Faculty of Science, Azhar University, Cairo, Egypt; 4 Physics Department, Faculty of Science, Fayoum University, Fayoum, Egypt

**Keywords:** Polyurethane foam, Nano-fillers samples, Radiation shielding, MCNP., Materials science, Physics, Applied physics, Nuclear physics, Techniques and instrumentation

## Abstract

This study investigates the potential of pure polyurethane (PU) foam as a lightweight, cost-effective shielding material against ionizing radiation, emphasizing its adaptability for incorporating high-performance fillers. PU foam was doped with various materials, including NiO, ZnO, Cr_2_O_3_, MnO_2_, BaO(Fe_2_O_3_)_6_, and sludge (at 44.5 wt.% loading), to enhance its shielding properties. The synthesized composites were characterized using Fourier Transform Infrared Spectroscopy (FT-IR), Scanning Electron Microscopy (SEM), and X-ray Fluorescence (XRF). Radiation shielding performance was evaluated through Monte Carlo simulations (MCNP) and Phy-X software for γ-rays (0.015–15 MeV) and fast neutron attenuation (up to 11 MeV). Results showed that incorporating high-density, high-atomic-number fillers significantly improved γ-ray attenuation, with BaO(Fe_2_O_3_)_6_ demonstrating the highest linear attenuation coefficient. Conversely, pure PU foam effectively attenuated fast neutrons due to its high concentration of light elements. The findings highlight PU-based composites as promising materials for γ-ray and neutron shielding, particularly in X-ray protection and radiological safety applications.

## Introduction

Gamma and X-rays are considered high-energy ionizing radiation that we cannot do without in many areas of our lives, whether in medical fields such as diagnosis using X-rays or nuclear medicine, as well as in industrial, agricultural, and security sectors in addition to that, research areas such as research reactors and nuclear power plants for electricity generation^1,2^. This increases the risk to public health, as excessive exposure to these rays can cause harm to human health^3^. The extent of the damage depends on the ionizing radiation the person is exposed to, as well as the duration of exposure, which can lead to the ionization of water within the cells that make up human tissues^4^. This can result in mutations in the cell’s function or death, which is considered an immediate effect^5^. Additionally, exposure to this radiation may damage the genetic fingerprint, leading to delayed effects that manifest in future generations^6,7^. To avoid the harm caused by ionizing radiation, we must search for materials that protect against these harmful radiations. To determine the appropriate shielding material, one must consider the weight of the material and its production cost^8,9^. The shielding material varies in density and atomic number depending on the type of radiation from which protection is sought. γ-rays are characterized by their high penetrating ability, so the material used as a shield against them must have a high density and a large atomic number to increase the likelihood of interaction between the γ-rays and this material, Whether it’s the photoelectric effect, compton scattering, or pair production^10–13^. Lead is considered one of the effective shields against γ-rays due to its high density and large atomic number; however, it is classified as a highly toxic material with chemical instability^14^.

As a result of the high cost and difficulty in shaping and maintaining heavy-density materials with a large atomic number used for protection against γ-rays, there has been a shift towards researching materials that are lower in production cost and more accessible to handle in terms of shaping and maintenance. These features are available in foam (polyurethane), in addition to being a sound and thermal insulating material. It can also be used as a matrix infused with compounds and elements to enhance its physical properties and increase its ability to protect against ionizing radiation^15^. In a previous attempt, lightweight, inexpensive materials with high flexibility were manufactured as radiation protection shields by adding Pb_3_O_4_ polyurethane in proportions of 60%, 66.6%, 75%, and 80%^16^. Experiments were also conducted on foam filled with water using different energies of γ-rays and a neutron source, and the values of the linear attenuation coefficient were greater than those of the foam filled with aluminum. Ni et al. created a one-step, laboratory-scale, fast-curing shielding material with a two-component polyurethane matrix and PbO filler. Increased filler content increased viscosity. Only a slight discrepancy existed between the components as filler content rose and curing time reduced. A minimum tack-free time of 27 s was attained at 70 wt.% filler. Boosted filler content initially boosted tensile and compressive strength, but it declined. Even with 60 wt% filler, mechanical characteristics were better than the matrix. Increased filler content lowered cohesive strength. At 60 wt.% filler, cohesion strength exceeded 100 kPa. Increased filler content enhances γ-ray shielding, whereas increasing composite thickness enhances performance under high γ-ray energy. Composites with 60 wt% filler had outstanding comprehensive characteristics^17^. M. M. Dorostkar et al. examined PU foams (PUF) for radiation shielding, noise, and heat resistance due to their lightweight, high resistance, and comfortable construction. This study examined the properties of lead oxide-doped polyurethane as a γ-shield through simulation and experimentation. Several lead weight fractions were used to sample the shield. ^137^Cs was used as the gamma source in both simulation and experiment using the MC code. The results show promise for gamma radiation attenuation. Ahmed M. El-Khatib et al. examined how adding Pb nano/microparticles to polyurethane foams improved thermo-physical and mechanical properties. In-situ polymerization creates a lead/polyurethane nanocomposite. Different characterization methods describe foam filler dispersion. These additions were tested in the foam using some techniques (e.g., FTIR, etc.) to analyze lead morphology and dispersion in polyurethane. The results show that lead is evenly distributed in polyurethane. The shielding efficiency, MAC and Z_eff_ were measured experimentally or by MC. The FLUKA code simulated nuclear radiation shielding for photon energies up to 100 MeV^18^. Mahdieh Mokhtari Dorostkar et al. simulated and tested a lead oxide-doped polyurethane gamma shield. Several lead weight fractions were used to sample the shield. ^137^Cs was used as the gamma source in both simulation and experiment using the MC-X2.6 Monte Carlo code. The results show promise for gamma radiation attenuation^19^.

In this study, innovative composite materials were developed by incorporating compounds NiO, ZnO, Cr_2_O_3_, MnO_2_, BaO(Fe_2_O_3_)_6_, and sludge into pure polyurethane to enhance its γ-ray attenuation properties. These compounds were specifically selected for their high atomic numbers and density, which are critical for effective radiation shielding using the Phy-X software and the Monte-Carlo (MC) simulation code. The samples comprise a 44.500% of NiO / MnO_2_ / ZnO / Cr_2_O_3_ / BaO(Fe_2_O_3_)_6 _/ sludge and 55.500 wt.% polyurethane while the seventh sample was pure polyurethane without any added compounds. The seven samples were synthesized and characterized via Fourier transform infrared spectroscopy (FT-IR) spectroscopy and Scanning Electron Microscopy analysis (SEM), as well as the X-ray Fluorescence (XRF) for the sludge nano-composites. A comparison was made between the pure foam sample and the other samples regarding their ability to attenuate γ-rays. The linear attenuation coefficient (LAC) for these samples was calculated, and the effectiveness parameters of these samples in protecting against ionizing radiation were represented by many factors. Also, the fast removal (FRCS) for the PU-X was discussed. The combination of diverse additives, systematic characterization, and theoretical verification highlights the novelty of this work and establishes a new benchmark for the development of lightweight, efficient radiation-shielding materials.

## Materials and methods

### Materials

**Fig. 1:**
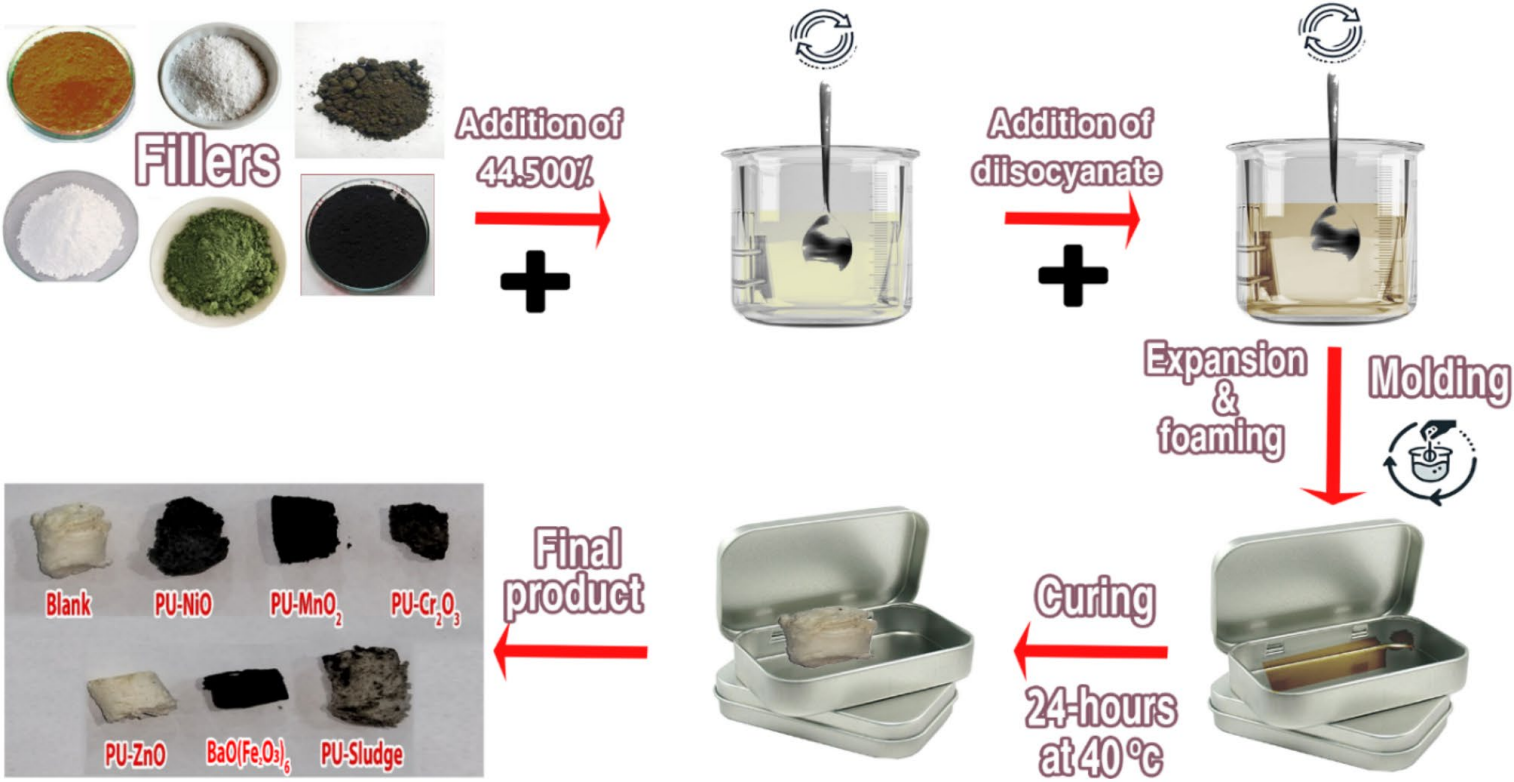
The preparation steps for PU-X composite foams.

**Fig. 2:**
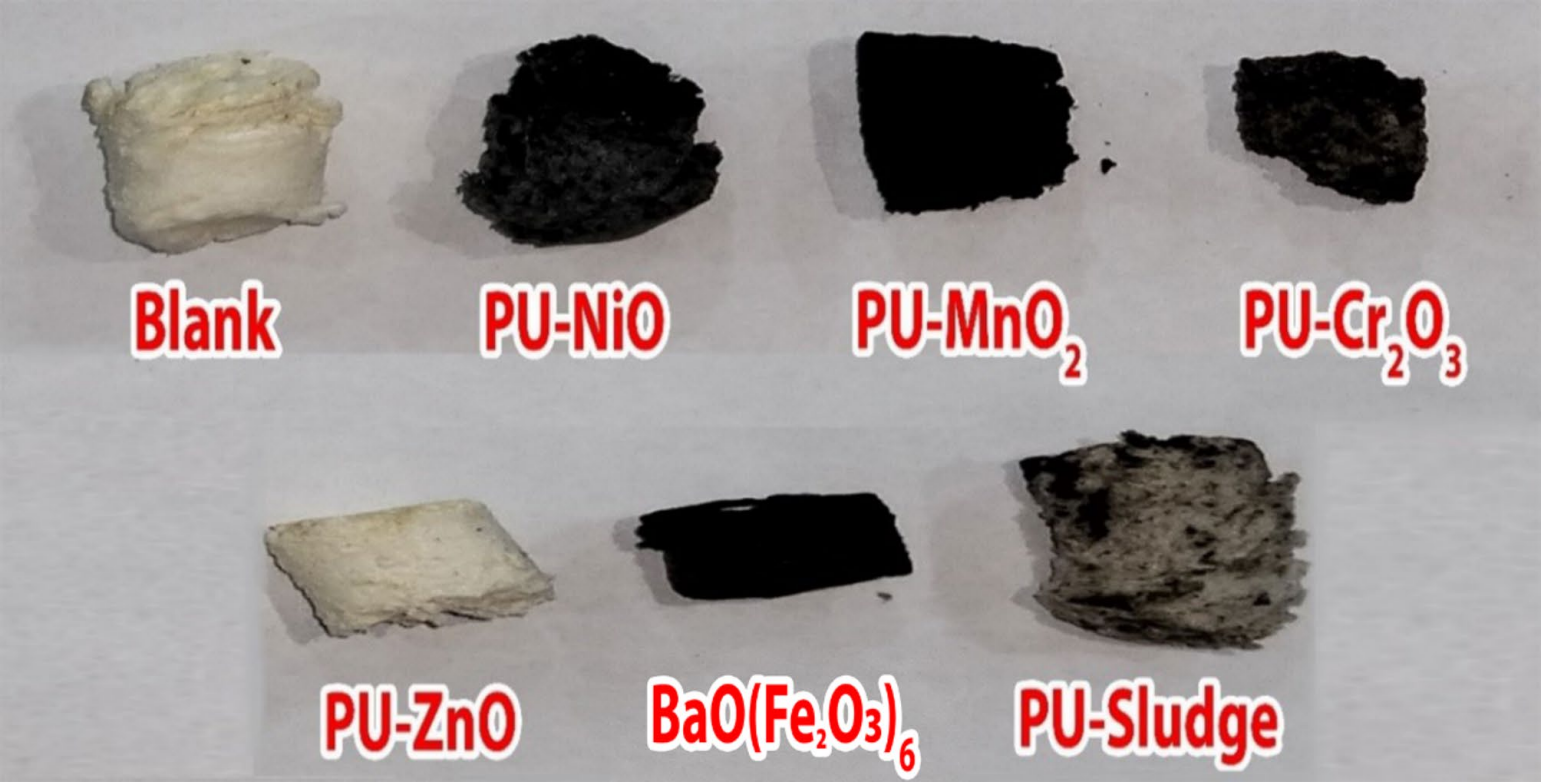
The prepared polyurethane foam composites PU-X (44.5 wt.%).

Seven composite foams comprising MnO_2_, NiO, Cr_2_O_3_, ZnO, BaO(Fe_2_O_3_)_6_, Sludge, and polyurethane (PU) were synthesized, featuring filler loadings of 44.5 wt.%. A 576-watt probe sonicator (model PRO250, Hoverlabs, USA) was utilized to disperse the fillers within the polyol for 10 min. Subsequently, the components were carefully blended in a 300 mL container using a wooden implement. Methylene diphenyl diisocyanate (MDI) was then incorporated into the polyol mixture at a molar ratio of 0.270:1.0 to promote polymerization. The composite mixture was allowed to cure overnight at room temperature. Figure 1 depicts the preparation steps for the PU composite foams. Samples’ code, chemical composition, and density of the prepared PU-X foam samples are listed in Table 1. The prepared PU-X foam samples are presented in Fig. 2.

**Table 1:** The chemical oxides composition of the prepared PU-X foam samples.

Samples code	Composition (Wt. %)							Density, (g.cm^− 3^)
	PU	NiO	MnO_2_	ZnO	Cr_2_O_3_	BaO(Fe_2_O_3_)_6_	Sludge	
PU-Blank	100	–	–	–	–	–	–	0.0524 ± 0.002
PU-NiO	55.500	44.500	–	–	–	–	–	0.0911 ± 0.350
PU-MnO_2_		–	44.500	–	–	–	–	0.1500 ± 0.270
PU-ZnO		–	–	44.500	–	–	–	0.0740 ± 0.006
PU-Cr_2_O_3_		–	–	–	44.500	–	–	0.1091 ± 0.010
PU-BaO(Fe_2_O_3_)_6_		–	–	–	–	44.500	–	0.1551 ± 0.012
PU-Sludge		–	–	–	–	–	44.500	0.1120 ± 0.011

### Methods

#### Characterization and instrumentations

For this investigation, several different characterization methods were utilized in order to investigate the characteristics of the assembled PU-X nano-composite samples. FT-IR spectroscopy was employed to analyze the chemical structural changes induced by incorporating the different fillers into the PU matrix (BaO(Fe_2_O_3_)_6_, Cr_2_O_3_, MnO_2_, NiO, Sludge, and ZnO). The morphological properties of the PU and PU composites (MnO_2_@PU, NiO@PU, Cr_2_O_3_@PU, ZnO@PU, BaO(Fe_2_O_3_)_6_@PU, and Sludge@PU foams were examined using a Zeiss EVO-10 SEM from Germany. Also, the XRF analysis was used to know the elemental composition of the used sludge material. To ensure the materials were analyzed in their original, unaltered form, the polymeric foam specimens were cryogenically fractured using liquid nitrogen before examination. The instruments’ specifications are outlined in Table 2.

**Table 2:** Instrumentations’ specifications that characterize the prepared PU-X foam samples.

Instrument name	Model	Data	Conditions	Technique
Fourier-Transform-Infrared Spectrophotometer (FT-IR)	BRUKER Tensor 37	FT-IR spectrum	400–4000 cm^− 1^	KBr pellets
Scanning electron microscope (SEM)	JSM-lT200, JEOL Ltd	SEM images	Imaging mode	Sputtering coating (J-E-O-L-JFC-1100E)
X-ray Fluorescence (XRF)	Shimadzu lab x 6100, Kyoto, Japan	XRF Analysis	10 ppm	X-U-Q Helium

#### Density determination

Measurements were taken with the Ultra-Pycnometer (1000), Quanta-chrome Upyc 1200-e-V-5.04 under room temp. Conditions were used to determine the densities of the PU-X foam samples that were put through the testing process^20^. With the help of gas movement, this apparatus makes it possible to perform accurate calculations of the structural or apparent density of materials that are either solid or particle-based. This technique uses helium gas to determine the amount of visible porosity present in the sample. A substance’s volume is measured to determine its apparent density. This is accomplished by introducing a particular quantity of gas into a chamber at two different pressures throughout the experiment. The volume is calculated using the equation P_1_V_1_ = P_2_V_2_^21^.

#### Radiation shielding investigation

##### MC simulation

**Fig. 3:**
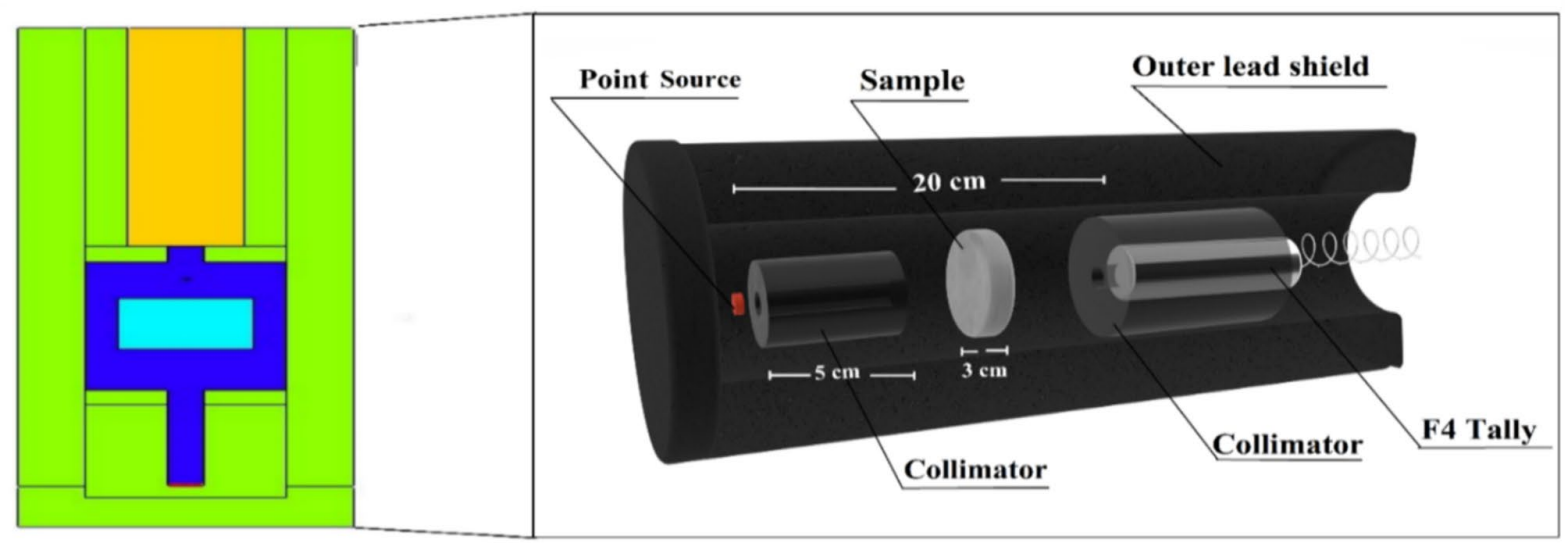
The MC-simulation used for studying PU-X foam samples.

To predict the γ-radiation density from sources at γ-energies (Eγ) ranging from 0.015 to 15 MeV, a simulation code was used, supplemented with the necessary data such as the distances between the source, sample, and detector, as well as the dimensions of the source and detector, in addition to the chemical composition and density of the samples under study^22,23^. The objective is to liken the intensity of γ-rays before and after traversing the PU-X foam samples under investigation. MC codes are frequently favored in research studies, encompassing radiation protection and safety, dose calculations, detector design, and more^24^. These preferences are ascribed to various advantageous attributes inherent in these symbols, including their capacity to function across a broad energy spectrum, adaptability to diverse geometric configurations, and swift computational capabilities^25^. The technique’s purpose is to facilitate the undertaking of neutrons and γ-rays, considering several mechanisms for photon interaction^26^. To perform a MC simulation, it is imperative to furnish precise details in the input file concerning the geometry, the distance from the source to the detector, the dimensions of the source (SDEF card), and the elemental/chemical structure and densities of the samples under investigation. The simulation’s geometric configuration was established using a preliminary two-dimensional and three-dimensional setup, as illustrated in Fig. 3. All criteria have been considered to ensure adherence to the experimental system. The input files for the MC simulation have been formatted as text files^27^. The cell comprised a separate component: a radiation source, primary and secondary γ-radiation modifiers, a cubic sample, and a detector. The chosen radioactive source was positioned behind a lead collimator located 20 cm from the detector. The neutron source is a fission spectrum, functioning within a photon energy range of up to 12 million electron volts to swiftly diminish cross sections. The samples were constructed as cylindrical layers and positioned far from the source and the detector. The detector was placed within a lead collimator intended for secondary γ-rays. The matter computes the total values within the range F4:P, which determines the average path length of the γ  photons generated by point simulation sources. A lead external shield was employed to encase the detector, source, and samples under examination. The LAc (µ, cm^− 1^) can be calculated using the equation:^10,28–31^

1 $$I\,=\,{I_o}{e^{ - \mu x}}$$

The half-tenth-mean free values (*HVl*,* TVl*,* and* MFp) can be estimated as follows:

2 $$\:HVL,\:cm\:=\:\frac{0.693}{LAc},\:\:$$

3 $$\:TVL,\:cm=\:3.323\:HVL\:\:$$

4 $$\:MFP,\:cm=\:1.443\:HVL$$

When analyzing the potential attenuation provided by various shielding materials, it is important to consider the radiation protection efficiency (RPe). It is possible to ascertain the RPe, % factor by performing the calculations provided below^32,33^:

5 $$\:RPe,\:\%=(1-\:\frac{I}{{I}_{o}})100$$

It was determined explicitly that a neutron source exists within the η-energy range, which extends up to 12 MeV. To assess the effectiveness of the fast neutron removal process, the tally F4: N was utilized. In every computation, the NPS value of 12 million particles per run is used to guarantee that statistical errors are minimal. The fast neutron removal cross-section (FRCS, Σ_R_ cm^− 1^) of PU-X samples is a parameter commonly used to describe the neutron-slowing characteristics of these samples. Quantification of the interaction between photons and matter is achieved through the linear attenuation coefficient. The removal of fast neutrons by materials can be compared to this process, denoted by the symbol Σ_R_, cm^− 1^. In addition, the subsequent equations were utilized to determine the half-value layer (HVL_FRCS_, cm) and relaxation length (λ_FRCS_, cm) for the materials, which were derived from neutron calculations. The term “relaxation length” refers to the average distance a highly energetic neutron can travel before it comes into contact with the medium^34–36^.

6 $$\:{HVL}_{{\Sigma\:}_{\text{R}},}cm\:=\:\frac{ln2}{{\Sigma\:}_{\text{R}}},$$

7 $$\:{\lambda\:}_{{\Sigma\:}_{\text{R}},}\text{c}\text{m}=\frac{1}{{\Sigma\:}_{\text{R}}},$$

##### Phy-X software

An online software that provides values for LAC, HVL, TVL, MFp, Z_eff_, and other necessary parameters to theoretically evaluate the materials under test^37^. The atomic weight of the entire sample is calculated by determining the atomic weight of each element individually within an Excel sheet. The weight and the density, which have been previously calculated for each sample, are then entered. Next, the energy range of the γ-rays for which the shielding parameters need to be calculated is specified, and the results appear in the Excel sheet. This site is used because it offers a wide range of capabilities^38^. In the subsequent step, the µ values obtained from PhyX were compared with those derived from MC simulations to assess the relative variances (∆, %) as described in the previous Sect. 

8 $$\Delta \left( \% \right)=\left| {\frac{{MC - PhyX}}{{MC}}} \right| \times 100$$

## Results and discussion

### Structural characterizations

#### FT/IR spectroscopic

**Fig. 4:**
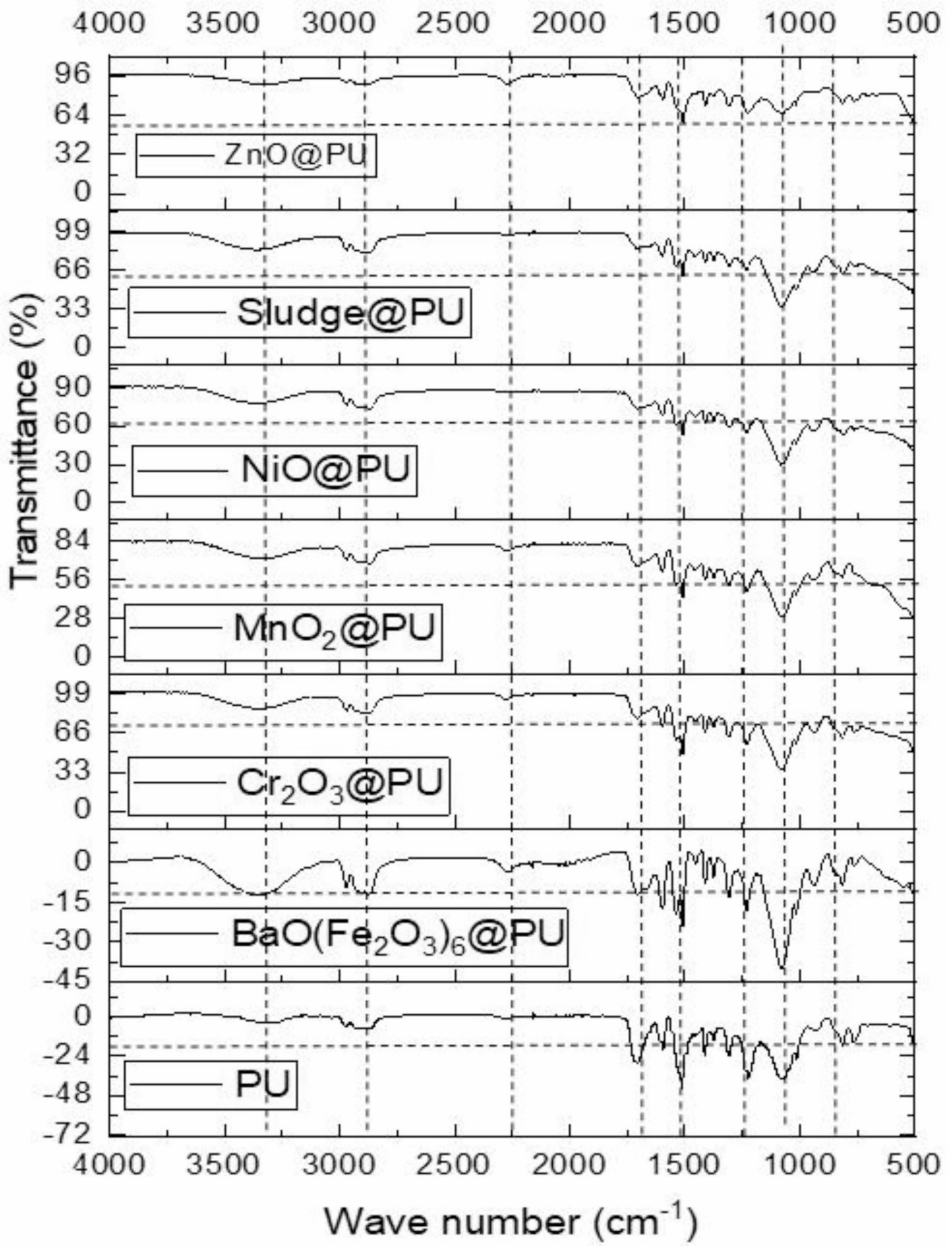
The FT-IR spectra of pure PU and PU-X composite foams.

Fig. 4 illustrates the FT-IR spectra of pure PU and PU/fillers (BaO(Fe_2_O_3_)_6_, Cr_2_O_3_, MnO_2_, NiO, Sludge and ZnO). In the pure PU foam, the characteristic bands at 3427 cm^−1^ and 3520 cm^−1^ correspond to the stretching vibrations of hydrogen-bonded and free (N–H) groups, respectively^39^. The absorption bands at 2930 cm^−1^ and 2866 cm^−1^ are attributed to the asymmetric and symmetric stretching vibrations of CH_2_ groups. Additionally, the peaks observed at 1722 cm^−1^, 1228 cm^−1^, and 1072 cm⁻¹ are associated with carbonyl (C = O) stretching, aromatic (C–O) stretching, and asymmetric (C–O–C) stretching vibrations, respectively^40^. The combined (N–H) deformation and (C–N) stretching vibrations are evident at 1533 cm^−1^ and 1228 cm^−1^^41,42^. The near disappearance of the peak at 2270 cm^−1^ signifies the depletion of the isocyanate (–NCO) group during the polymerization process, while the appearance of the imine (C = N) band at 1600 cm^−1^ confirms this change^43^. Examining the FT-IR spectra of neat PU and PU/fillers reveals that the relative positions of the characteristic peaks in the composite foams are relatively comparable to those of the pure PU foam^44^. Thus, the FT-IR analysis suggests that adding different fillers does not significantly alter the chemical structure of the segmented PU foam^45^.

#### Morphological studies

**Fig. 5:**
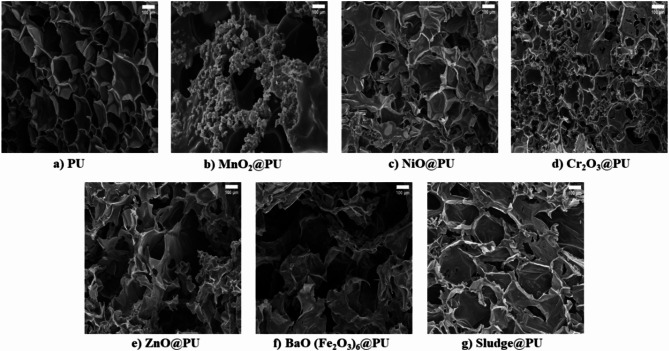
SEM micrographs for PU and PU composite foams.

Fig. 5 demonstrates the SEM micrographs of the PU and PU composite foams (MnO_2_@PU, NiO@PU, Cr_2_O_3_@PU, ZnO@PU, BaO(Fe_2_O_3_)_6_@PU, and Sludge@PU) provide critical insights into the morphology and dispersion of filler materials within the polymer matrix, influencing the composites’ effectiveness in shielding gamma radiation. The average pore size for the seven samples is (310, 270, 240, 210, 207, 200 and 170) µm for PU, MnO_2_@PU, NiO@PU, Cr_2_O_3_@PU, ZnO@PU, BaO(Fe_2_O_3_)_6_@PU, and Sludge@PU respectively. Fig. 5a illustrates the SEM image of the pristine PU revealed a smooth, homogeneous surface, typical of neat polymers^45,46^. The average pore size is 310 μm. Fig. 5b shows the SEM image of the MnO_2_@PU composite foam. It shows a rougher surface with distinct MnO_2_ agglomerates. The filler particles are well embedded in the polymer matrix but show some degree of clustering. These clusters could act as scattering centers for γ-rays, contributing to the composite’s enhanced shielding ability. However, the formation of large agglomerates may create voids or defects, which could adversely affect mechanical properties. Fig. 5c demonstrates the NiO@PU composite foam which exhibited a more uniform dispersion of filler particles compared to MnO_2_. The NiO particles appear to be finer and more evenly distributed within the PU matrix, resulting in a composite with a relatively smoother surface compared to MnO_2_@PU. This uniform distribution is beneficial for optimizing the composite’s shielding efficiency, as it ensures consistent interaction with incident gamma rays across the entire material^47^. Fig. 5d illustrates the SEM micrograph of Cr_2_O_3_@PU which displayed a relatively porous structure with visible Cr_2_O_3_ particle agglomerations. Such porosity could potentially reduce the shielding capacity by introducing pathways for radiation to penetrate. However, Cr_2_O_3_’s high atomic number might still compensate for this to some extent, enhancing attenuation despite the structural imperfections^48^. Fig. 5e shows the ZnO@PU composite foam. It shows a highly dense and compact structure with ZnO particles well distributed throughout the matrix. The filler particles appear as bright spots on the SEM image, indicating their successful incorporation into the PU matrix without significant agglomeration. This dense structure is likely to contribute to improved gamma radiation attenuation due to ZnO’s high electron density and effective dispersion^49^. Fig. 5f displays the SEM image of the BaO (Fe_2_O_3_)_6_@PU composite, which revealed a complex structure with both BaO and Fe_2_O_3_ particles dispersed within the polymer. There is evidence of slight clustering, but the particles are relatively well incorporated into the matrix. The high atomic number of BaO and Fe_2_O_3_ makes this composite a strong candidate for gamma shielding, and the SEM micrograph suggests that the dispersion quality is sufficient to maximize interaction with gamma rays. However, localized particle agglomerations could influence shielding uniformity^50^. Fig. 5g illustrates the Sludge@PU composite foam, which presents a highly irregular surface morphology with a more heterogeneous distribution of the filler material. The presence of sludge particles creates significant roughness and discontinuities in the PU matrix, which could lead to both scattering and absorption effects. However, the non-uniform dispersion might result in areas of reduced shielding efficiency, depending on the filler’s composition and density 18. In comparing the seven samples, it is evident that the filler type and distribution play critical roles in determining the overall structure and potential shielding efficiency. Composites such as ZnO@PU, with its dense and uniform filler dispersion, and BaO(Fe_2_O_3_)_6_@PU, with high-density fillers, appear particularly promising for γ-radiation attenuation due to their homogeneity and high atomic numbers. On the other hand, MnO_2_@PU and Cr_2_O_3_@PU show some degree of aggregation and porosity, which could affect both mechanical properties and shielding performance. Sludge@PU, while offering a highly irregular surface, Sludge@PU may introduce unique scattering effects, but its heterogeneous distribution might limit its overall efficiency.

### XRF analysis

The composition of the sludge that was analyzed is presented in Table 3, along with the weight percentages of different components. The X-ray diffraction (XRF) analysis (Table 3) indicates that the sludge sample contains a significant amount of Fe_2_O_3_ (45.90 ± 0.25%). Additionally, the sample includes an assortment of other elements (e.g., Si, Ti, Ca, Al, Mg, etc.).

**Table 3:** The composition by (wt.%) of the sludge material.

Oxide	Wt.%	Element	Wt.%
Fe_2_O_3_	45.90 ± 0.25	Fe	32.10 ± 0.17
SiO_2_	25.79 ± 0.22	Si	12.06 ± 0.1
Al_2_O_3_	8.15 ± 0.14	Al	4.31 ± 0.07
CaO	7.65 ± 0.13	Ca	5.47 ± 0.09
TiO_2_	3.54 ± 0.09	Ti	2.12 ± 0.06
K_2_O	2.61 ± 0.08	K	2.17 ± 0.07
P_2_O_5_	2.35 ± 0.08	Px	1.02 ± 0.03
MgO	0.677 ± 0.034	Mg	0.408 ± 0.02
S	0.665 ± 0.033	S	0.665 ± 0.033
MnO	0.642 ± 0.032	Mn	0.498 ± 0.025
CdO	0.462 ± 0.08	Cd	0.404 ± 0.07
ZnO	0.329 ± 0.016	Zn	0.264 ± 0.013
SrO	0.156 ± 0.0078	Sr	0.132 ± 0.0066
ZrO_2_	0.154 ± 0.0077	Zr	0.114 ± 0.0057
Re_2_O_7_	0.127 ± 0.023	Re	0.0973 ± 0.017
V_2_O_5_	0.115 ± 0.0073	V	0.0642 ± 0.0041

### Radiation shielding investigation

#### Gamma rays’ attenuation

The linear attenuation values for the PU-X foam samples were presented using MC and PhyX for γ-energy ranging (Eγ) up to 15 MeV. There was a good agreement between the values obtained via MC code and those calculated by the Phy-X software with a maximum deviation of ∆ = 2.541% which confirm the γ-attenuation performance of the PU-X samples. As the γ-ray energy increases, the speed of the γ-photons also increases, leading to a decrease in the interaction cross-section (ϱ). Consequently, the probability of γ-rays interacting with the material decreases, leading to a reduction in the LAC for all samples.

The LAC values decline from 1.101 to 0.018 cm^− 1^ for PU-Blank, 25.411 to 0.023 cm^− 1^ for PU-NiO, 14.970 to 0.021 cm^− 1^ for PU-Mn_2_O, 14.716 to 0.022 cm^− 1^ for Cr_2_O_3_, 25.156 to 0.023 for PU-ZnO, 24.120 to 0.025 cm^− 1^ for PU-BaO(Fe_2_O_3_)_6_ and 10.474 to 0.021 cm^− 1^ for PU-Sludge at 0.015 ≤ Eγ ≤ 15 MeV.

**Fig. 6:**
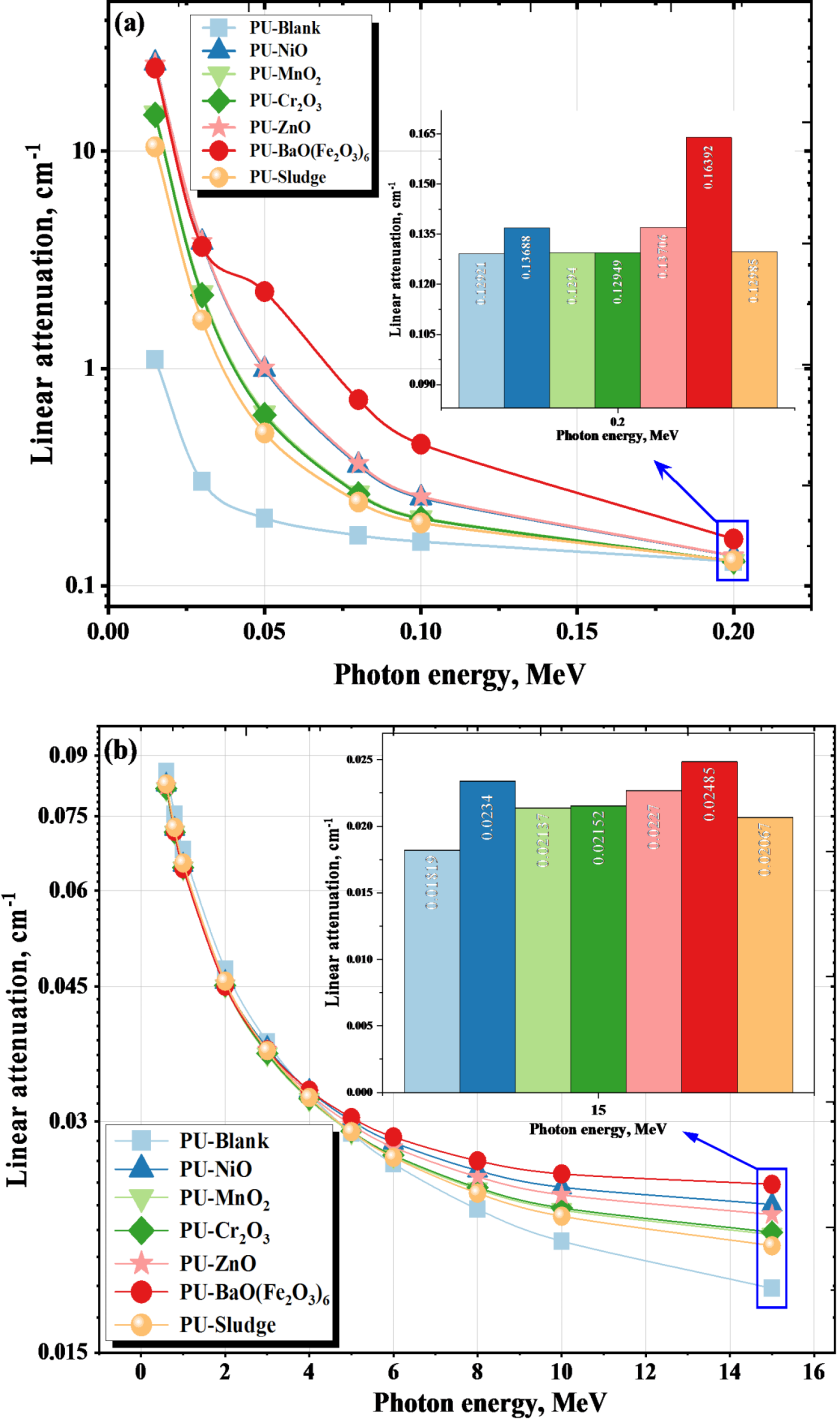
(**a**,**b**) The effect of γ-energy on the linear attenuation at (**a**) photoelectric, (**b**) compton scattering regions for the PU-X foam samples.

There was a sharp decrease in the simulated LAC values which were from 1.101 to 0.129 cm^− 1^ for PU-Blank, 25.411 to 0.137 cm^− 1^ for PU-Nio, 14.970 to 0.129 cm^− 1^ for Mn_2_O, 14.716 to 0.129 cm^− 1^ for PU-Cr_2_O_3_, 25.156 to 0.137 for PU-ZnO, 24.120 to 0.164 cm^− 1^ for PU-BaO(Fe_2_O_3_)_6,_ and 10.474 to 0.130 cm^− 1^ for PU-Sludge at 0.015 ≤ Eγ ≤ 0.200 MeV. That was because of the photo-electric (PE) interaction with the PU-X foam samples as shown in Fig. 6a where ϱ is proportional to E^− 3:−5^^51–53^.

There was a slight smooth drop in the LAC values from 0.113 to 0.018 cm^− 1^ for PU-Blank, 0.112 to 0.023 cm^− 1^ for PU-Nio, 0.109 to 0.021 cm^− 1^ for PU-MnO_2_, 0.109 to 0.022 cm^− 1^ for PU-Cr_2_O_3,_ 0.112 to 0.026 for PU-ZnO, 0.120 to 0.025 cm^− 1^ for PU-BaO(Fe_2_O_3_)_6_ and 0.110 to 0.021 cm^− 1^ for PU-Sludge at 0.3 ≤ Eγ ≤ 15 Mev because of the compton (CE) interaction as shown in Fig. 6b (ϱ is proportional to E^− 1^)^53,54^. In these samples, we note that pair production (PP) interaction’s probability remains lower than the compton probability even at Eγ ≤ 15 MeV^55,56^.

The addition of high-density fillers (e.g., sludge and BaO(Fe_2_O_3_)_6_) and transition metal oxides contributes significantly to enhancing the attenuation, as these materials promote increased interaction probabilities due to their higher effective atomic numbers and densities. The highest value of the LAC found in the PU- BaO(Fe_2_O_3_)_6_ sample due to the high atomic number of Ba and Fe specially in the PE effect range^57,58^. In contrast, the lowest value is in sample PU-Blank due to the low atomic number of the elements content .

**Fig. 7:**
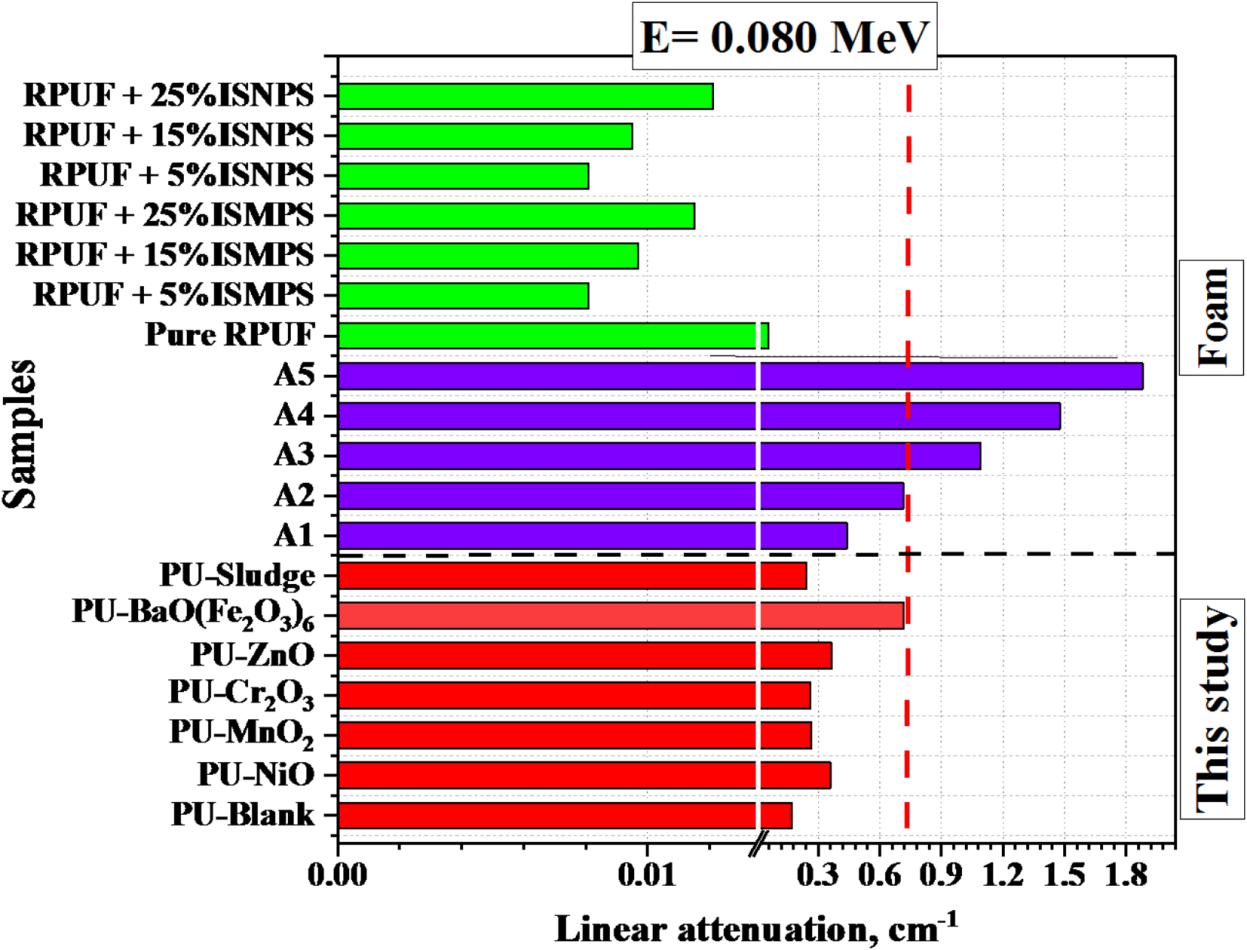
Comparison of linear attenuation at E = 0.080 MeV across diverse foam materials for the PU-based composites.

Fig. 7 presents a comparison of the LAC for the PU-X foam samples and two sets of previously published foam samples at selected energy Eγ = 0.080 MeV (Common energy used in X-ray applications). The first Set consists of rigid polyurethane (RPUF) composite samples with different proportions of iron slag as additives (RPUF + X-ISN/MPS) where X = 0 − 25% in increments of 5%. The second set comprises composites foam filled with mixtures of Epoxy(60%) + B_2_O_3_(20%) + CaO(20%-X) + CeO_2_(X) where X = 0–20 with step size of 5%^59^.

The PU-X foam samples were found higher than those A1, A2, and RPUF + X-ISN/MPS foam samples and lower than the epoxy-based samples; A3, A4, and A5 samples at 0.080 MeV as seen in Fig. 7.

**Fig. 8:**
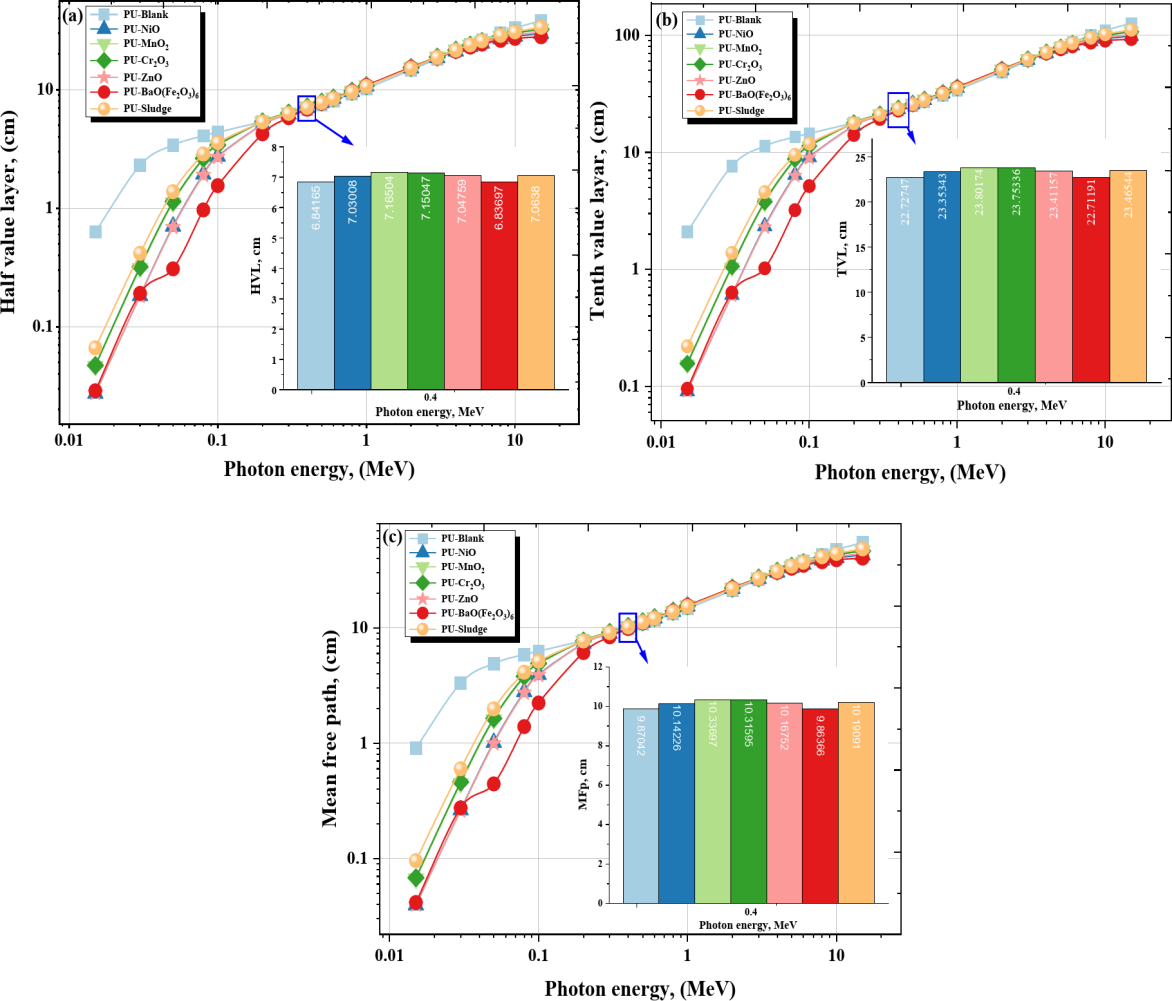
(**a**) The HVL, (**b**) The TVL, and (**c**) The MFP for the PU-X foam samples vs. the γ-energy.

To design protective shields from γ-rays, the HVL and TVL values ​​of the PU-X foam samples were studied to determine the ideal thickness of these samples that achieves the highest attenuation of γ-rays. The values of HVL and TVL are inversely proportional to the linear attenuation coefficient^60,61^. The HVL increased from 0.629 cm to 38.105 cm for blank, 0.027 cm to 29.627 cm for NiO, 0.046 cm to 32.443 cm for MnO_2_, 0.047 cm to 32.202 cm for Cr_2_O_3_, 0.028 cm to 30.535 cm for ZnO, 0.029 cm to 27.892 cm for BaO(Fe_2_O_3_)_6_ and 0.066 cm to 33.536 cm for Sludge. The highest value of HVL is found in the Blank sample (in the X-rays region, 0.015 to 0.200 MeV) because it has the lowest value of the µ, the lowest HVL value is found in the BaFe_6_ sample because it has the highest value of the linear attenuation as shown in Fig. 8a. The same principle is taken for TVL Fig. 8b^62^. MFp is the average path that γ-rays travel within the material before they interact. MFP is the reciprocal of LAC, so the higher the MFP values, the lower the LAC values^63,64^. The MFP were ranged from 0.908 cm to 54.974 cm for Blank, 0.039 cm to 42.742 cm for NiO, 0.067 to 46.805 cm for MnO_2_, 0.068 cm to 46.458 cm for Cr_2_O_3_, 0.040 cm to 44.053 cm for ZnO, 0.041 cm to 40.240 cm for BaO(Fe_2_O_3_)_6_ and 0.095 cm to 48.382 cm for Sludge foam sample. The highest value of MFP is found in the pure sample because it has the lowest value of the linear attenuation, the lowest MFP value is found in the BaO(Fe_2_O_3_)_6_ sample because it has the highest value of the linear attenuation at γ-energy up to 0.200 MeV shown in Fig. 8c.

**Fig. 9:**
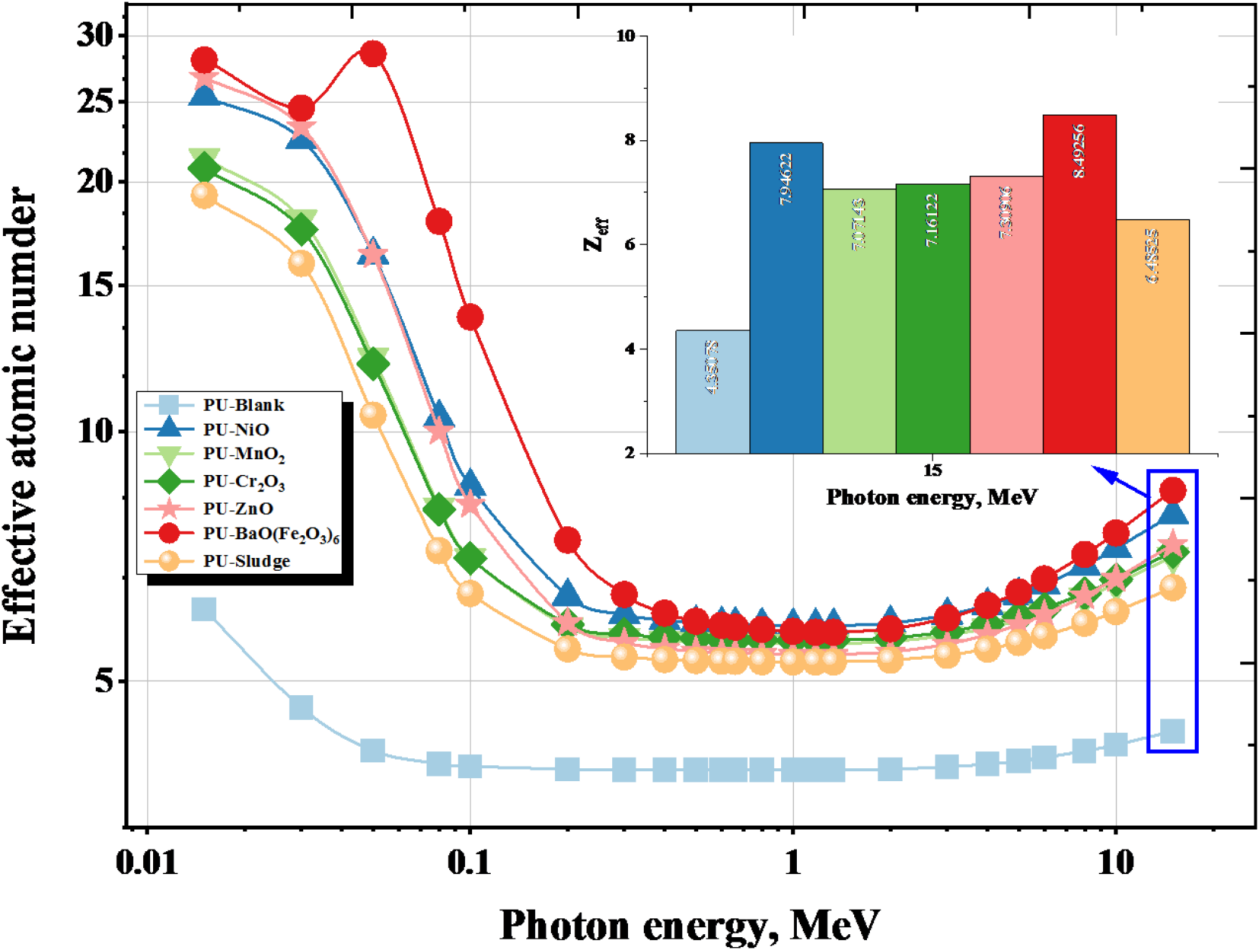
The effective atomic number for the PU-X foam samples with the γ-energy.

To evaluate protection against γ-rays, the Z_eff_ of the materials used must be calculated, as it indicates the number of electrons contributing to the interaction between γ-rays and the atom^65^. The Z_eff_ values decrease with increasing γ-energy from 0.015 MeV to 15 MeV these values fall from 6.102 to 4.351 for Blank, 25.387 to 7.946 for NiO, 21.401 to 7.071 for MnO_2_, 20.760 to 7.161 for Cr_2_O_3_, 26.754 to 7.309 for ZnO, 28.085 to 8.493 for BaO(Fe_2_O_3_)_6_ and 19.274 to 6.485 for Sludge. The highest value of Z_eff_ is found in the BaO(Fe_2_O_3_)_6_ sample, and the lowest Z_eff_ value is found in the Blank sample shown in Fig. 9. The results show that the BaO(Fe_2_O_3_)_6_ sample has ideal determinants to be used as an additive to foam for protection against γ-rays.

**Fig. 10:**
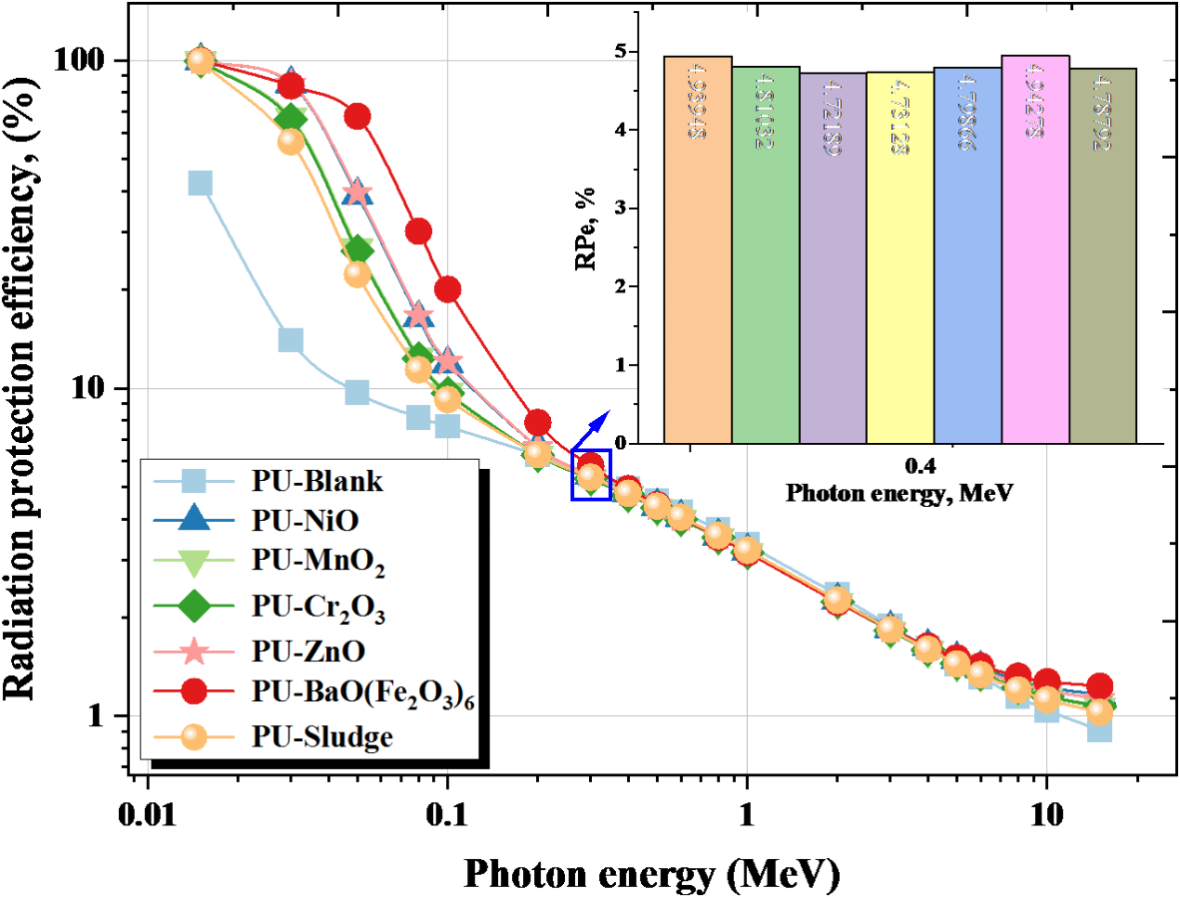
The RPe for the PU-X foam samples vs. the γ-energy.

Fig. 10 illustrates the RPe for the PU-X foam samples influenced by γ and the incorporation of fillers. The RPe approaches approximately 100% at very low γ (up to 0.015 MeV). As the γ increased, the penetrating strength of the emitted γ also escalated, resulting in a notable decrease in the RPe, %. As the γ-electron interaction intensifies, the number of scattered γs escalates, adversely affecting the RPe for the PU-X foam samples. The RPe decreased by 0.015 MeV from approximately 99.999% for the PU-X samples to 11.910%, 9.744%, 9.673%, 12.046%, 20.065%, and 9.247% for the PU-NiO, PU-MnO_2_, PU-Cr_2_O_3_, PU-ZnO, PU- BaO(Fe_2_O_3_)_6_, and PU-Sludge samples, respectively, at γ = 0.100 MeV. The findings validate a significant shielding efficacy of the PU-X foam with its fillers in the γ-range of 0.015 to 0.100 MeV. Conversely, the PU-Blank exhibits the lowest RPe. The PU- BaO(Fe_2_O_3_)_6_ exhibits the highest RPe % among the synthesized PU-X, attributable to its elevated concentrations of Ba and Fe elements, which increases photoelectric absorption and improves linear attenuation coefficient (LAC) by providing more atoms for γ-ray interactions. Together, these properties enhance γ-ray attenuation, particularly in low- and mid-energy ranges, making it an effective lightweight radiation shielding solution.

From the previous results, these materials exhibit superior γ-ray attenuation properties compared to pure polyurethane, making them suitable for various radiation protection scenarios. Moreover, The PU-based composites developed offer a lightweight alternative that can be easily integrated into building materials for hospitals, nuclear facilities, and other radiation-prone environments without compromising structural integrity. Also, it makes them ideal for use in personal protective equipment, such as radiation shielding garments and gloves for medical and nuclear industry workers. The incorporation of these materials could lead to more comfortable and ergonomic designs due to their reduced weight. In addition, it can be fabricated into portable panels or barriers for use in temporary shielding applications, such as during maintenance work in radiation zones or as emergency shielding in nuclear incidents. Their flexibility and ease of handling provide significant advantages over conventional materials. Also, these composites can be customized to provide optimal shielding performance for specific radiation energy ranges. This versatility is critical for applications such as medical imaging (e.g., X-rays, CT scans) or industrial radiography. Finally, the incorporation of industrial waste materials like sludge not only enhances the shielding properties but also provides an eco-friendly solution for waste management. This approach aligns with sustainability goals by reducing environmental impact and repurposing waste into high-value materials.

#### Neutron shielding investigation

**Fig. 11:**
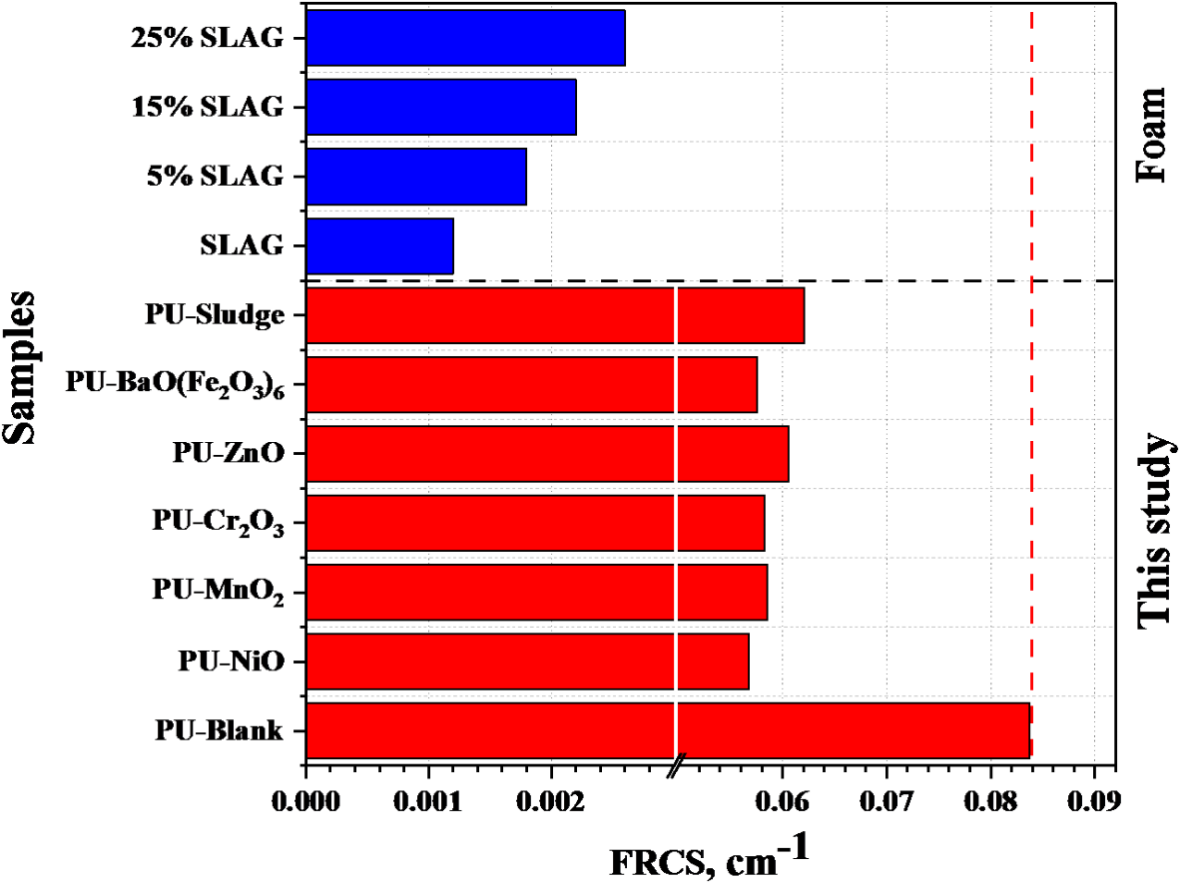
Comparison of the fast neutron removal cross-section (Σ_R_) for the investigated PU-X foam and some other published.

**Fig. 12:**
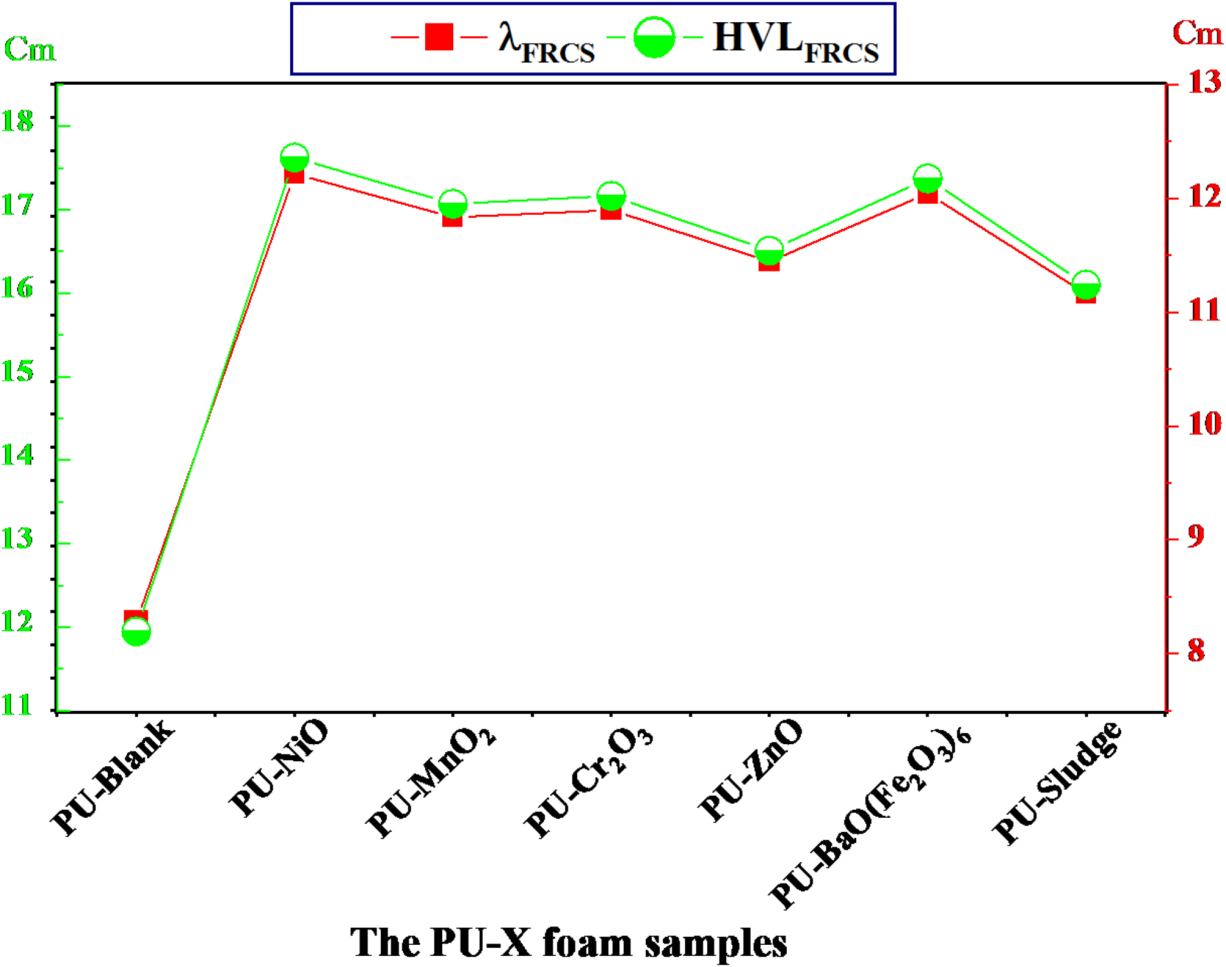
The λ_FRCS_ and the HVL_FRCS_ for the investigated PU-X foam samples.

As seen in Fig. 11, the PU-X foam’s Σ_R_ values were compared with some other published foam rigid polyurethane (RPUF) composite samples with different proportions of iron slag as additives coded as X-SLAG where X = 0%, 5%, 15%, 25%^66^. The values of the Σ_R_ were found 0.084, 0.057, 0.059, 0.058, 0.061, 0.0576, and 0.062 cm^− 1^ for PU-Blank, PU-NiO, PU-MnO_2_, PU-Cr_2_O_3_, PU-ZnO, PU-BaO(Fe_2_O_3_)_6_, and PU-Sludge sample, respectively. The PU-X foam samples were found higher than those compared due to the high contented of the light elements (e.g., O, C, N, etc.). Also, Fig. 12 represent the HVL_ΣR_, cm and λ_ΣR_, cm for the PU-X foam samples which reflect the inverse effect of the Σ_R_.

## Limitations and future studies

The study has several limitations that warrant further investigation. It evaluated a limited selection of fillers (e.g., BaO(Fe_2_O_3_)_6_, NiO, ZnO), leaving room to explore other high-density materials and novel nanomaterials for improved performance. The focus was primarily on γ-rays and fast neutrons, without assessing the foam’s effectiveness against other ionizing radiation types, such as alpha or beta particles, which may restrict its applicability. Additionally, the mechanical properties of the doped PU foam, such as durability, flexibility, and wear resistance, were not thoroughly examined, though these are critical for practical use. The findings are largely based on laboratory and simulation results, emphasizing the need for real-world testing under diverse environmental conditions, such as varying temperature and humidity. Finally, the study used a fixed filler concentration (44.500 wt%), leaving the effects of varying concentrations on both shielding efficiency and structural integrity unexplored.

Future research should explore additional filler materials by investigating other high-density compounds, metallic oxides, or advanced nanocomposites to further enhance radiation shielding performance. Alongside this, the mechanical and thermal properties of doped PU foam should be examined to verify its suitability for practical applications across various environmental conditions. The impact of varying filler concentrations and combinations warrants further study to optimize the balance between shielding efficiency, weight, and structural integrity. Moreover, it is crucial to investigate the potential of PU foam.

## Conclusion

Pure polyurethane (PU) foam, due to its lightweight, cost-effectiveness, and ease of fabrication, is a promising material for shielding against ionizing radiation. To enhance its radiation attenuation properties, PU foam was doped with high-density, high-atomic-number fillers, including NiO, ZnO, Cr_2_O_3_, MnO_2_, BaO(Fe_2_O_3_)_6_, and sludge at a loading of 44.5 wt.%. These composites were synthesized and analyzed for their shielding capabilities using Phy-X software and Monte Carlo simulations (MCNP). Results demonstrated that the addition of fillers significantly improved the linear attenuation coefficient (LAC) of PU foam, particularly at lower γ-ray energies. The BaO(Fe_2_O_3_)_6_ sample showed the highest LAC values, especially in the X-ray energy range up to 0.100 MeV, attributed to the presence of high-atomic-number elements (Ba and Fe). While LAC decreased with increasing γ-ray energy for all samples, the doped composites consistently outperformed pure PU foam. This study confirms the potential of PU-based composites as effective radiation shielding materials for X-ray and γ-ray protection applications.

## Data Availability

All data generated or analyzed during this study are included in this published article.
